# Spontaneous Pneumomediastinum in a Patient With Asthma: A Case Report

**DOI:** 10.7759/cureus.113502

**Published:** 2026-07-28

**Authors:** Diptee Poudel, Ingrid Estrada Ariza, Kathryn Lang, Christine Hyun, Roxana Lazarescu

**Affiliations:** 1 Internal Medicine, Wyckoff Heights Medical Center, New York, USA

**Keywords:** asthma, copd, oxygen support, s: pneumomediastinum, spontaneous

## Abstract

Pneumomediastinum is described as free air located within the mediastinum. In the absence of traumatic injury, iatrogenic injury, or a clear etiology, it is called spontaneous pneumomediastinum (SPM). SPM has been associated with many conditions and triggers, including bronchial asthma, forceful straining during exercise, inhalation of drugs, and activities associated with the Valsalva maneuver. This case details a 25-year-old female patient with a past medical history of asthma who presented to the emergency department with cough, shortness of breath, and chest pain. Initial chest X-ray demonstrated subcutaneous emphysema of the lower neck, and subsequent CT revealed extensive pneumomediastinum. The patient was admitted and treated for an acute asthma exacerbation with bronchodilators, corticosteroids, and analgesics. Supplemental oxygen was administered to facilitate nitrogen washout, and serial chest radiographs were obtained to monitor for progression or development of pneumothorax. The patient’s respiratory symptoms improved, pulmonary function stabilized, and follow-up imaging demonstrated interval improvement of the pneumomediastinum without complications. This case highlights the importance of recognizing SPM as a potential complication of asthma exacerbations and emphasizes the role of supportive management and monitoring.

## Introduction

Spontaneous pneumomediastinum (SPM) is described as free air located within the mediastinum that can occur in the absence of trauma and may be associated with factors that increase intrathoracic pressure, including asthma, chronic obstructive pulmonary disease, forceful emesis or coughing, chronic airway irritation from smoking, and strenuous physical activity [1,2]. It is usually a self-limiting condition, but serious complications should be monitored for, such as tension pneumomediastinum or, rarely, tension pneumothorax [1]. While treatment is aimed at the precipitating factor, the severity of these potential complications proves the necessity of effective treatment for the pneumomediastinum itself. The following case report outlines the clinical progression and management of an asthmatic patient who developed a pneumomediastinum as a result of an acute asthma exacerbation.

## Case presentation

**Figure 1:**
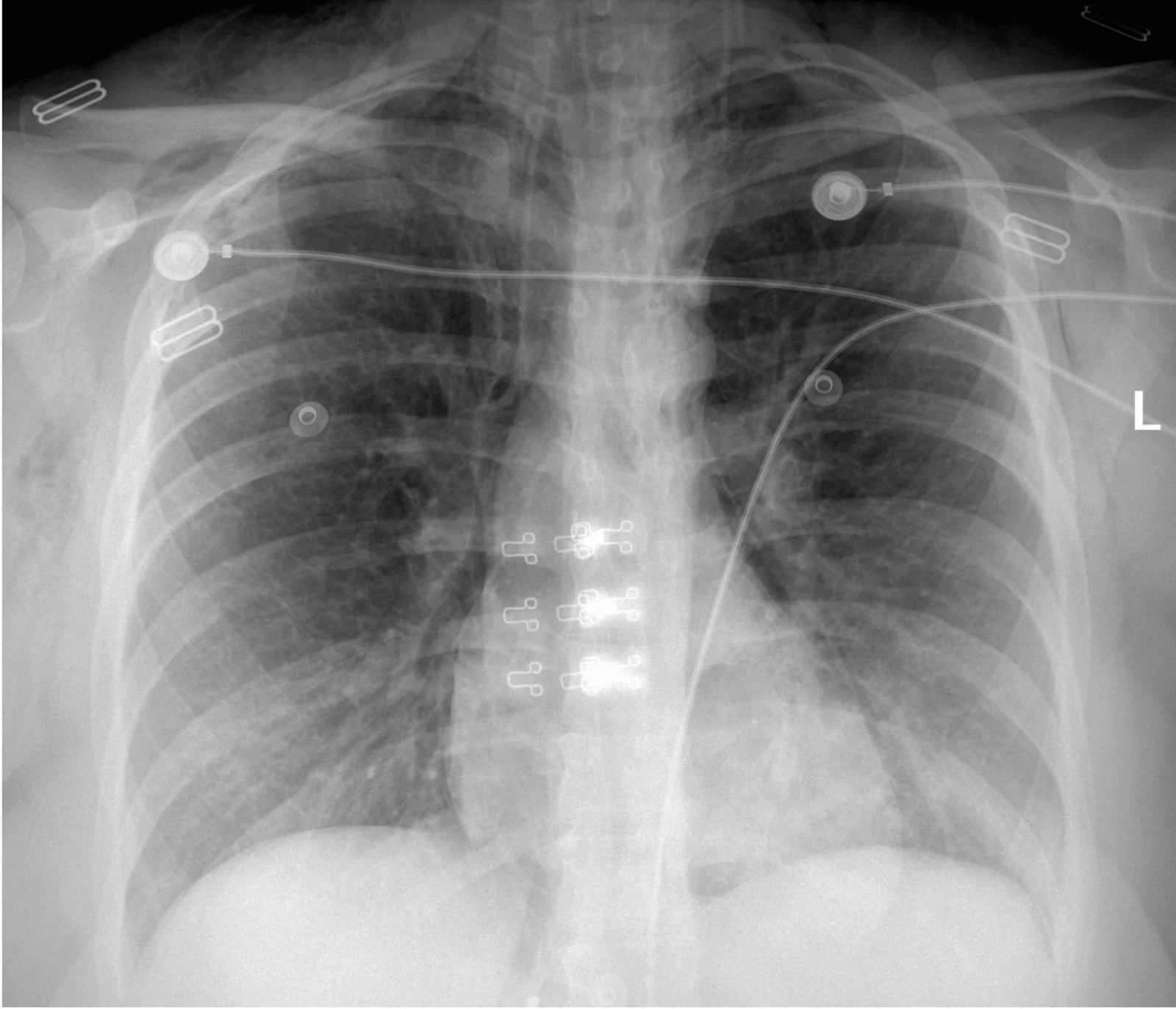
X-ray of the chest AP view showing subcutaneous gas across the lower neck associated with pneumomediastinum and pneumopericardium.

**Figure 2:**
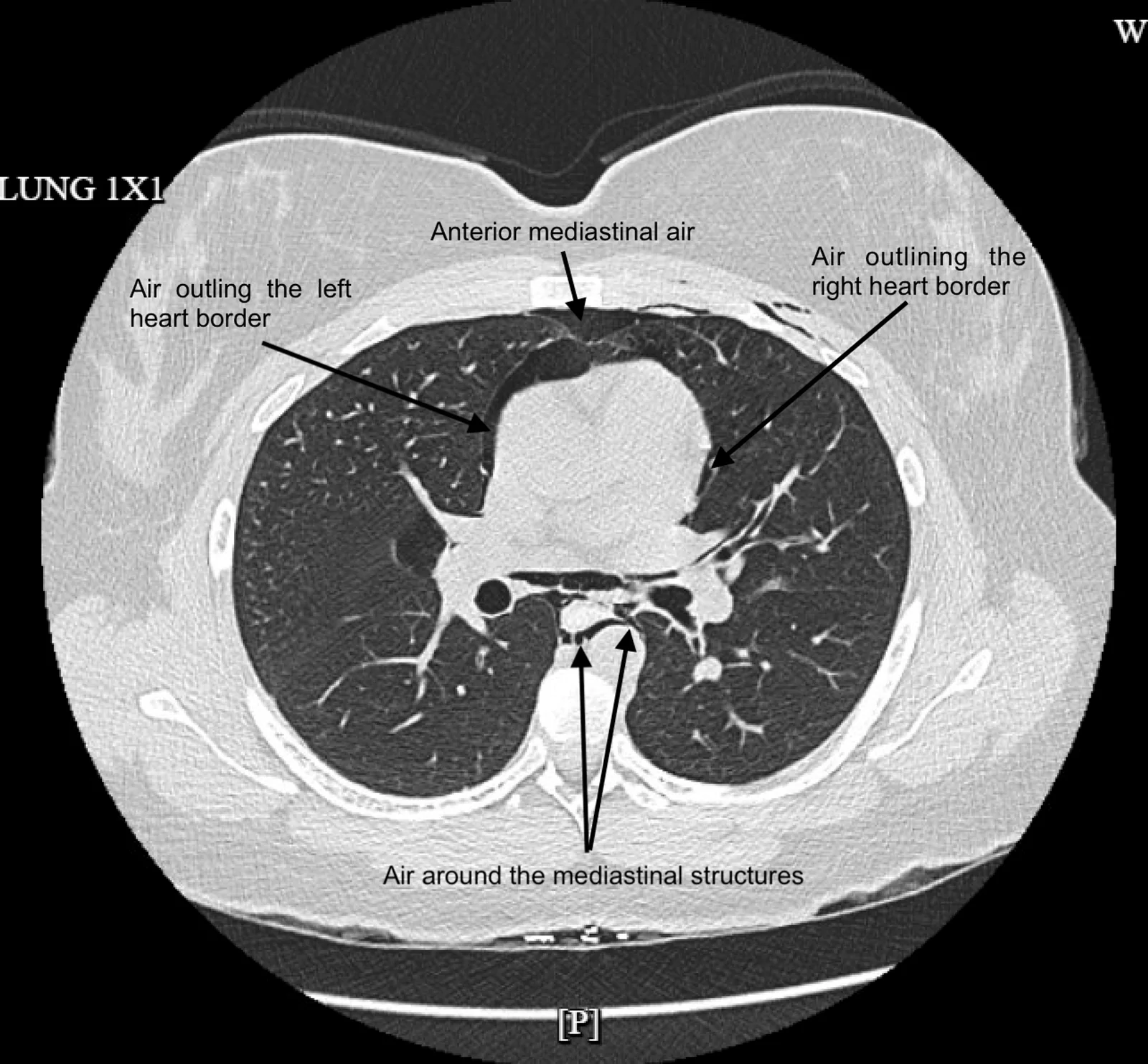
CT chest without contrast showing extensive pneumomediastinum with small pneumopericardium anteriorly and extensive subcutaneous emphysema in the anterior thorax.

A 25-year-old female patient with a medical history of asthma presented to the emergency department with a cough, shortness of breath, and chest pain worse with inspiration. Her symptoms began the day prior and initially improved with her albuterol inhaler but worsened the following day again with no improvement with the inhaler. She reports coughing vigorously. Of note, she was a non-smoker, and her asthma had remained clinically well controlled since childhood with as-needed albuterol and Trelegy inhalers and intermittent montelukast therapy, without recent exacerbations. She was tachycardic at a rate of 140 s and normotensive with bilateral lung wheezes and upper chest crepitus when presenting to the emergency department and was sent for chest X-ray, which showed subcutaneous gas across the lower neck (Figure 1). CT chest showed extensive pneumomediastinum with small pneumopericardium anteriorly and extensive subcutaneous emphysema in the anterior thorax (Figure 2). The patient was admitted and treated for an acute asthma exacerbation, evidenced by tachycardia (heart rate 140 beats/min), tachypnea (respiratory rate 22 breaths/min), and a reduction in peak expiratory flow to 230 L/min from a baseline of approximately 400 L/min (57.5% of personal best). She was treated with inhaled levalbuterol and ipratropium bromide every four hours, inhaled budesonide every 12 hours, intravenous methylprednisolone sodium succinate 60 mg every six hours with gradual tapering, and montelukast 10 mg daily. In addition to protocol-directed management of the patient’s acute asthma exacerbation, the patient received oxygen via a non-rebreather mask to wash out the nitrogen.

**Figure 3:**
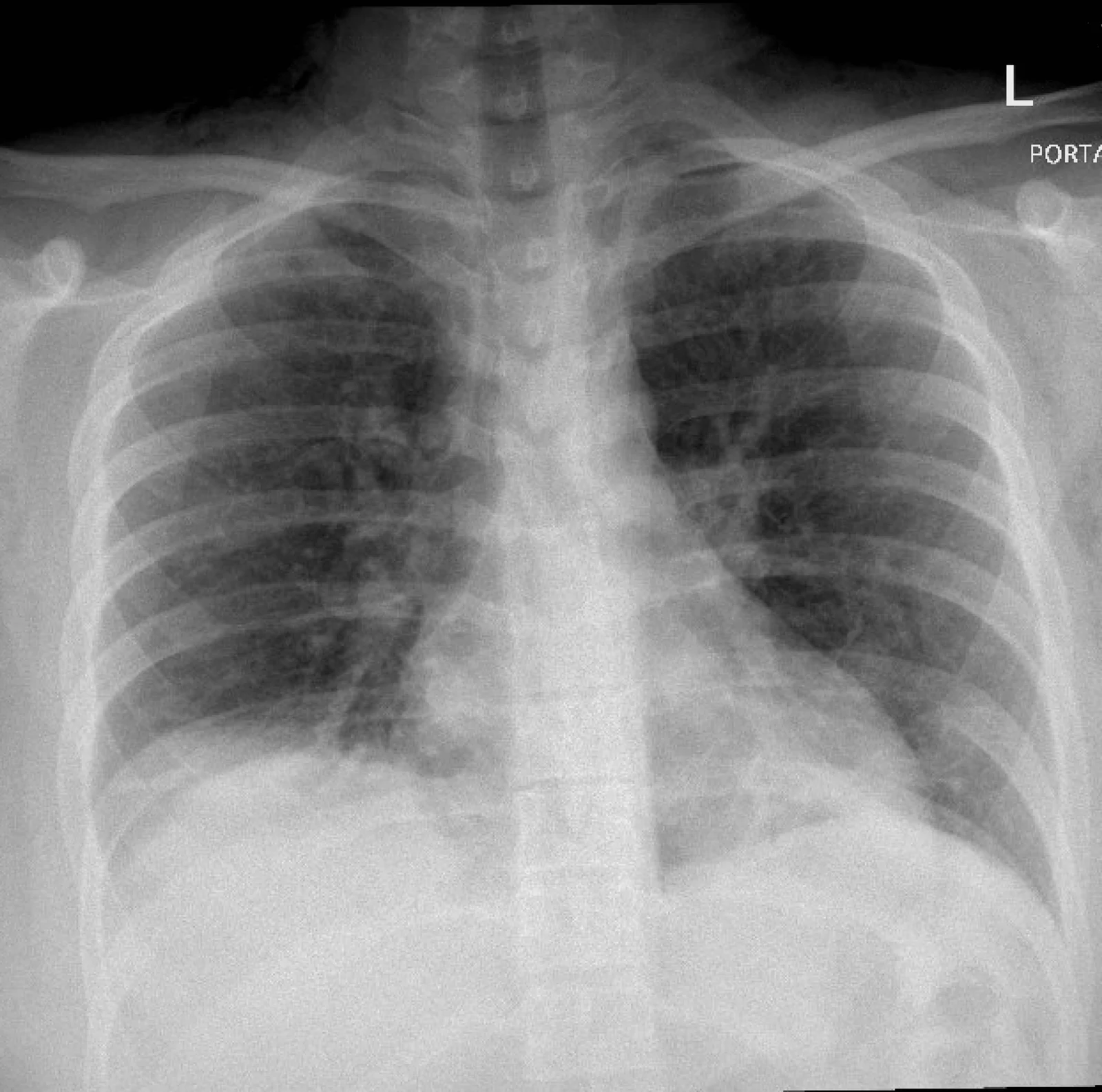
Chest X-ray demonstrating interval improvement of the pneumomediastinum, with persistent residual right paratracheal pneumomediastinum and pneumopericardium.

Her clinical symptoms improved after treatment. Repeat chest X-rays were performed to monitor for expansion of the pneumomediastinum into a pneumothorax. Peak flow measurements improved to 400 L/min. Follow-up chest radiograph obtained on hospital day 3 demonstrated interval improvement of the pneumomediastinum, with a small amount of persistent right paratracheal mediastinal air (Figure 3). The patient was discharged with home albuterol, ipratropium, pantoprazole, prednisone, and budesonide/formoterol.

**Figure 4:**
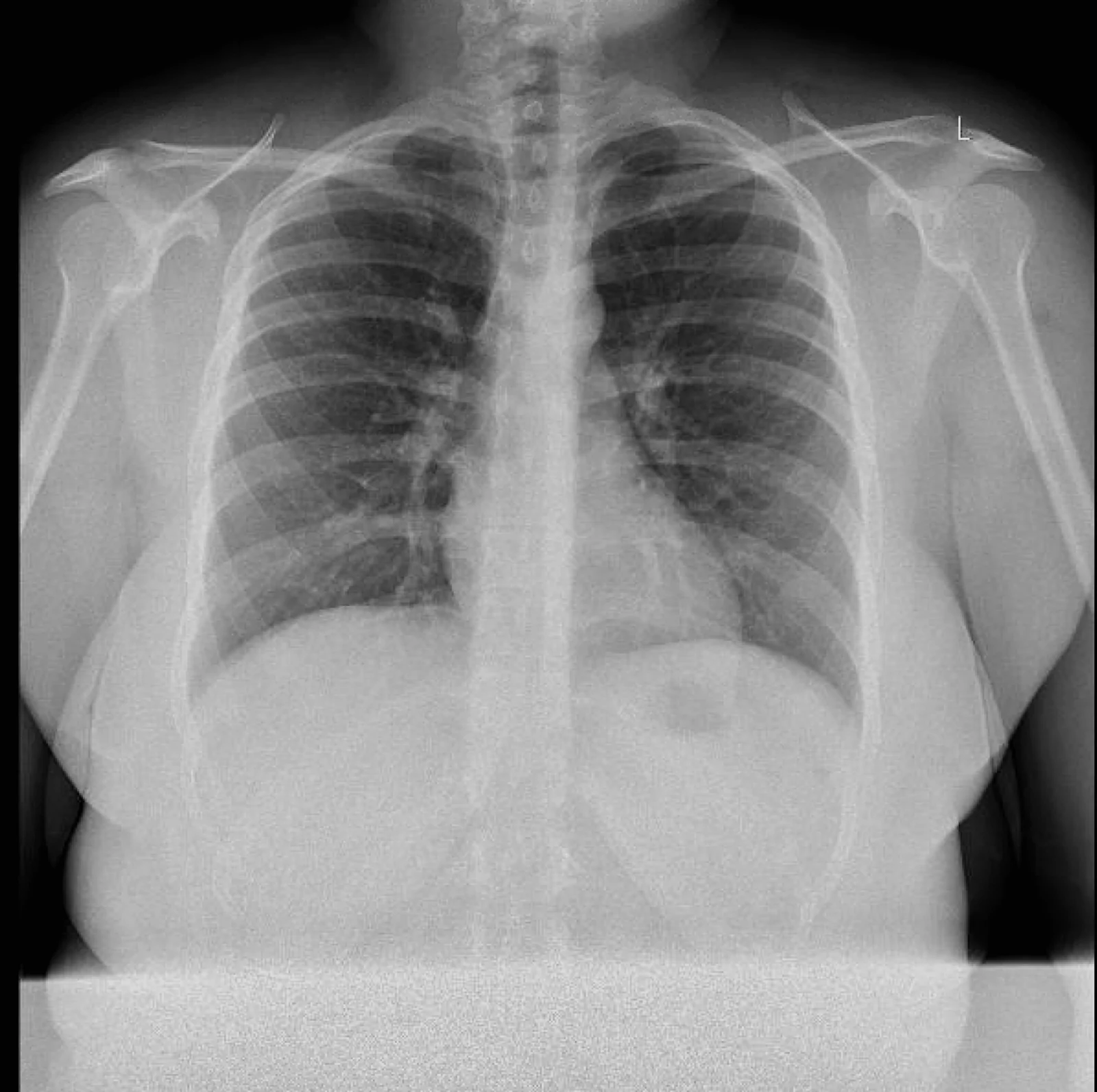
Follow-up posteroanterior (PA) chest X-ray demonstrating interval improvement of the pneumomediastinum, with a small residual right paratracheal pneumomediastinum. No pneumothorax is identified.

At the 10-day follow-up in the pulmonary clinic, the patient reported complete resolution of symptoms, including dyspnea, chest pain, cough, and neck discomfort. Follow-up chest radiography demonstrated near-complete resolution of the previously noted pneumomediastinum, with no evidence of recurrent pneumothorax or other acute cardiopulmonary abnormality (Figure 4).

## Discussion

SPM is an uncommon but recognized complication of acute asthma exacerbations, particularly in younger patients with forceful coughing and increased intrathoracic pressures [1]. The underlying pathophysiology of SPM is best explained by the Macklin effect, in which intense elevations in alveolar pressure can lead to alveolar rupture, allowing air to dissect along peribronchial and perivascular sheaths into the mediastinum [2]. This mechanism has been demonstrated in both clinical and imaging studies, where linear collections of air along bronchovascular bundles on CT are characteristic of this process [2].

Asthma is a well-documented predisposing factor for SPM. Large case series and recent reports have shown that patients with poorly controlled asthma or severe exacerbations are at increased risk of developing pneumomediastinum, likely due to repetitive and forceful coughing that significantly elevates intrathoracic pressures [3,4]. In one 2025 case series of eight asthmatic patients with SPM with an average age of 21, all exhibited severe exacerbations with extensive mediastinal air confirmed on chest CT, reinforcing the clinical association between asthma severity and SPM [4]. Similar case reports describe SPM in patients with asthma presenting with acute dyspnea, cough, chest pain, and subcutaneous emphysema, with favorable outcomes under conservative management [5].

Although SPM typically follows a benign clinical course, its signs and symptoms frequently overlap with other acute thoracic conditions such as pneumothorax and esophageal perforation, making diagnostic imaging crucial. CT imaging not only confirms the presence of mediastinal air but can also help identify signs of the Macklin effect seen in SPM and rule out other life-threatening etiologies [6,7]. Clinical vigilance is important because rare complications such as pneumopericardium, tension pneumomediastinum, or even tension pneumothorax can occur, particularly in the setting of severe respiratory compromise [7].

Management of SPM has traditionally been supportive. Strategies include supplemental oxygen to promote resorption of free air, analgesia for pain control, and treatment of any underlying triggers such as bronchospasm during asthma exacerbations [3,8]. Most studies support conservative management with bronchodilator therapy, corticosteroids, clinical observation, and serial chest imaging, as invasive interventions including chest tube placement, pericardiocentesis, or surgical intervention are rarely necessary in uncomplicated cases [3]. Emerging evidence also suggests that, in carefully selected stable patients, outpatient management may be feasible, thereby avoiding unnecessary hospitalization while still ensuring patient safety [9]. However, criteria for outpatient versus inpatient management have yet to be standardized and should be determined on a case-by-case basis considering patient stability and comorbidity burden [9].

Long-term outcomes for patients who experience SPM in the context of asthma appear favorable, with low rates of recurrence noted in retrospective analyses. However, preexisting lung disease, such as asthma, may be associated with a slightly increased risk of recurrence compared with otherwise healthy individuals, highlighting the importance of optimal chronic disease management [6,10].

In this patient’s case, the combination of supportive care, including bronchodilators, corticosteroids, and supplemental oxygen, led to clinical improvement and resolution of the pneumomediastinum, as seen on chest X-ray radiographs. Avoidance of invasive diagnostic procedures and prophylactic antibiotics aligns with contemporary practice recommendations [11].

## Conclusions

SPM is a rare but meaningful complication of acute asthma exacerbation, particularly in young adults presenting with cough, dyspnea, and chest pain. Recognition of the characteristic radiographic and clinical features, especially when associated with the Macklin effect, allows clinicians to differentiate SPM from other critical thoracic emergencies. While the condition is often self-limiting, supportive management remains the cornerstone of treatment, emphasizing control of the underlying asthma exacerbation and careful observation for potential progression. Recent evidence supports conservative care with supplemental oxygen and monitoring in stable patients, and questions the necessity of routine hospitalization or prophylactic interventions in every case (self-limiting, supportive management remains the cornerstone of treatment, emphasizing control of the underlying asthma exacerbation and careful observation for potential progression). Clinicians should also be cognizant of the potential for recurrence in patients with underlying lung disease, reinforcing the importance of long-term asthma control.

## References

[1] Sahni S, Verma S, Grullon J, Esquire A, Patel P, Talwar A (2013). Spontaneous pneumomediastinum: time for consensus. N Am J Med Sci.

[2] Murayama S, Gibo S (2014). Spontaneous pneumomediastinum and Macklin effect: overview and appearance on computed tomography. World J Radiol.

[3] Talwar A, Rajeev A, Rachapudi S, Khan S, Singh V, Talwar A (2024). Spontaneous pneumomediastinum: a comprehensive review of diagnosis and management. Intractable Rare Dis Res.

[4] Biborchi H, Ajdir L, Bounhar S, Ijim M, Fikri O, Amro L (2025). Spontaneous pneumomediastinum complicating severe asthma exacerbation. World J Adv Res Rev.

[5] Caceres M, Ali SZ, Braud R, Weiman D, Garrett HE Jr (2008). Spontaneous pneumomediastinum: a comparative study and review of the literature. Ann Thorac Surg.

[6] Kumeda H, Saito G (2022). Spontaneous pneumomediastinum diagnosed by the Macklin effect. J Surg Case Rep.

[7] Iteen AJ, Bianchi W, Sharman T (2026). Pneumomediastinum. StatPearls [Internet].

[8] Susai CJ, Banks KC, Alcasid NJ, Velotta JB (2024). A clinical review of spontaneous pneumomediastinum. Mediastinum.

[9] Esme H, Gürbüzler ÖM (2025). Management of spontaneous pneumomediastinum: are hospitalization and prophylactic antibiotic treatment necessary?. Genel Tıp Derg.

[10] Yu MH, Kim JK, Kim T, Lee HS, Kim DK (2023). Primary spontaneous pneumomediastinum: 237 cases in a single-center experience over a 10-year period and assessment of factors related with recurrence. PLoS One.

[11] Ebina M, Inoue A, Takaba A, Ariyoshi K (2017). Management of spontaneous pneumomediastinum: are hospitalization and prophylactic antibiotics needed?. Am J Emerg Med.

